# Rehabilitation of a misbehaving microbiome: phages for the remodeling of bacterial composition and function

**DOI:** 10.1016/j.isci.2022.104146

**Published:** 2022-03-23

**Authors:** Hiba Baaziz, Zachary Robert Baker, Hollyn Claire Franklin, Bryan Boen Hsu

**Affiliations:** 1 Department of Biological Sciences, Virginia Polytechnic Institute and State University, Blacksburg, VA 24061, USA

**Keywords:** Virology, Microbiome

## Abstract

The human gut microbiota is considered an adjunct metabolic organ owing to its health impact. Recent studies have shown correlations between gut phage composition and host health. Whereas phage therapy has popularized virulent phages as antimicrobials, both virulent and temperate phages have a natural ecological relationship with their cognate bacteria. Characterization of this evolutionary coadaptation has led to other emergent therapeutic phage applications that do not necessarily rely on bacterial eradication or target pathogens. Here, we present an overview of the tripartite relationship between phages, bacteria, and the mammalian host, and highlight applications of the wildtype and genetically engineered phage for gut microbiome remodeling. In light of new and varied strategies, we propose to categorize phage applications aiming to modulate bacterial composition or function as “phage rehabilitation.” By delineating phage rehab from phage therapy, we believe it will enable greater nuance and understanding of these new phage-based technologies.

## Graphical abstract

**Figure:**
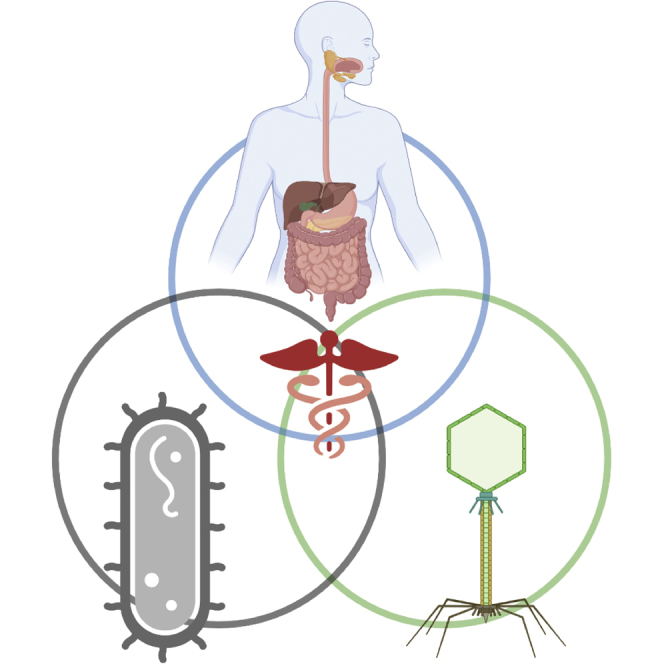

## The gut microbiome is significant to host health

The human holobiont comprises a diverse microbial community (microbiota) with a composition and density linked to numerous aspects of human health and disease. The gastrointestinal (GI) tract is the densest microbially colonized region of the body with more than 100 trillion microorganisms including viruses (prokaryotic and eukaryotic), bacteria, and at lower densities, archaea, fungi, and protozoa (Thursby and Juge, 2017; Turnbaugh et al., 2007). Partly owing to a high colonization density and limited resources available, these microbes can be highly interactive with an intricate and often symbiotic relationship with the mammalian host (Bäckhed et al., 2005; Ley et al., 2008; Thursby and Juge, 2017). Bacteria are among the most dominant human gut microbes at nearly 10^14^ cells (Sender et al., 2016) and generally have a commensal relationship with the host (Fan and Pedersen, 2021). However, genetic or environmental factors can result in a maladaptation of the gut microbiome that is deleterious, a state sometimes referred to as dysbiosis. The bacterial composition in the gut has been associated with a variety of host phenotypes including metabolic diseases (Fan and Pedersen, 2021), inflammatory diseases (Clemente et al., 2018), susceptibility to enteric infection (Ducarmon et al., 2019), and neurological disorders (Cryan et al., 2020), among others. Despite considerable advancement in our understanding of the relationship between the gut microbiome and disease, much remains to be characterized (Schmidt et al., 2018).

**Figure 1:**
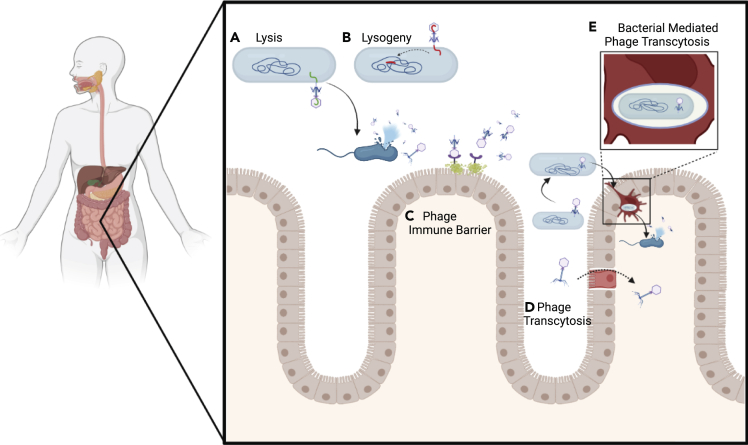
Mechanisms of phage interaction with bacteria and the mammalian host (A) Infection of bacteria with virulent phage leads to cell lysis and the release of progeny phage. (B) Temperate phage infection of a host bacterium can lead to the lytic life cycle or genomic integration into the bacterial chromosome as a prophage and the lysogenic life cycle. (C) Phage can attach to the mucosa through interactions with the capsid. (D and E) (D) Phage can cross the intestinal epithelium through transcytosis or (E) within a bacterial cell.

Compared with bacteria in the gut microbiota, non-bacterial microbes are understudied despite their potential significance. Bacterial viruses, also known as bacteriophages or phages, are obligate intracellular parasites that rely on the metabolic machinery of their bacterial hosts for propagation. Discovered over a century ago, phages are considered the most abundant and diverse biological entity inhabiting Earth’s ecosystems, with an estimated global population size of 10^31^ particles (Brüssow and Hendrix, 2002; Suttle, 2005; Twort, 1915). Whereas the composition and abundance of phages can vary along the GI tract, especially in the colon (Shkoporov and Hill, 2019), gut phages are estimated to be as numerically dominant as bacteria with ∼10^9^–10^10^ viral-like particles (VLP) per gram of stool (Hoyles et al., 2014). Though VLP counts include both prokaryotic and eukaryotic viral particles, the latter are comparatively rare (Minot et al., 2011). Furthermore, phages depend on their bacterial hosts for continued persistence. This leads to an interesting bidirectional relationship where fluctuations in one can alter the concentration and/or functions of the other (Weitz, 2016). The longitudinal relationship between phages and bacteria has been of considerable interest, especially in marine environments, leading to a number of ecological models. For example, in kill-the-winner dynamics, the virulent phage (i.e., infection leads to cell lysis and the release of the progeny phage) targeting an abundant bacterial species rapidly reduces the concentration of this cognate host (Figure 1A) (Thingstad, 2000), leading to the expansion of bacterial competitors (Maslov and Sneppen, 2017). These enriched competitors are in turn targeted by their own phages, resulting in fluctuations in the overall abundance and diversity of phage and bacterial communities (Thingstad, 2000). Which bacteria benefit and fill this vacated niche has been subject to additional modeling. A royal family model suggests that a subset of competing species adapted for the same niche will fill the vacancy, whereas an equal opportunity model indicates that this expansion will come from one of the other more numerous, yet less well-adapted, species (Breitbart et al., 2018). In the human infant gut microbiome, virulent phages are prevalent with observed kill-the-winner dynamics (Lim et al., 2015), whereas in the adult microbiome, lysogeny is more prevalent, complicating the application of this model. Accounting for temperate phages, the piggyback-the-winner model indicates that lysogeny (i.e., bacteria with a genomically integrated prophage) is beneficial in high-density microbial environments (Figure 1B), which can lead to higher levels of phage enrichment than that possible by lytic replication alone (Silveira and Rohwer, 2016).

### Microbiota transplantation can improve host health

The gut microbiota is a factor in various physiological functions such as energy harvesting through extraction, synthesis, and absorption of nutrients and metabolites (Den Besten et al., 2013), immunomodulation (Belkaid and Hand, 2014; Gensollen et al., 2016), and resistance to pathogen colonization (Sassone-Corsi and Raffatellu, 2015). In some instances of chronic disease associated with a compositionally maladapted gut microbiome, restoration of microbial richness and diversity can be achieved by fecal microbiota transplantation (FMT). FMT is a process in which bacteria, viruses, and other fecal components are extracted from healthy donor feces and administered to patients with depleted gut microbiomes (Broecker et al., 2016). This procedure has shown remarkable success in treating recurrent *Clostridioides difficile* infections (rCDI) (Van Nood et al., 2013). FMTs also have the potential for treating other diseases or disorders such as ulcerative colitis (Jacob et al., 2017), Crohn’s disease (Gordon and Harbord, 2014), chronic constipation (Tian et al., 2017), graft versus host disease (Van Lier et al., 2020), and neurodegenerative diseases (Wang et al., 2021). In instances where gut bacteria can alter drug efficacy, FMTs can reverse this effect as shown with microbiota-associated resistance to anti-PD-1 therapy (Davar et al., 2021). Whereas FMTs are extremely promising as a treatment, there are challenges owing to the use of donor material, which includes scalability issues and a costly reliance on screening for pathogenic species owing to its undefined nature (Panchal et al., 2018).

In addition to bacteria, other microbes such as fungi and phage are engrafted with long-term persistence (Zhang et al., 2021). For treating rCDI, recipients with lower *Caudovirales* richness and diversity compared with donors were all responsive to treatment and had significantly more *Caudovirales* taxa engrafted than in non-responders, although there were no significant differences in bacterial engraftment between responders and non-responders (Zuo and Ng, 2018). In another study where all rCDI patients responded to FMT treatment, no significant changes in *Caudovirales* richness and diversity were found; however, there were significant increases in the richness and diversity of *Microviridae* (single-stranded DNA phages) and bacteria post-FMT (Fujimoto et al., 2021). Moreover, the examination of the viral contigs transferred to recipients after FMT treatment for ulcerative colitis revealed a high frequency of genes associated with temperate phages, though it is unknown whether these were transferred as free viral particles or as prophages (Chehoud et al., 2016). On removing the potential effects of bacteria, transplantation of a fecal filtrate devoid of bacteria was sufficient to treat rCDI in a small cohort (*n* = 5) (Ott et al., 2017), which suggests that phages may play a causal role in the success of FMT. Fecal filtrates even showed superiority to conventional FMTs in preventing necrotizing enterocolitis in a preterm piglet model (Brunse et al., 2022). To further isolate the effect of a phage, conventionally colonized mice with diet-induced obesity (i.e., high-fat diet) were administered the viral particles from lean donor mice. This fecal virome transplantation (FVT) resulted in a partial resemblance of the recipient to the donor virome and increased the bacterial diversity but not the viral diversity, suggesting that even a partial engraftment of donor phages was substantial enough to drive phenotypic changes of reduced weight gain and improved glucose tolerance (Rasmussen et al., 2020). In another mouse study, antibiotic-treated conventional mice receiving an autochthonous FVT (i.e., donor material derived from the same mice before antibiotic treatment) showed gut microbiomes that more closely resembled a pre-antibiotic composition than mice receiving a heat-inactivated FVT (Draper et al., 2020). The success of FMT in treating rCDI has been a revelation for demonstrating the importance of a robust gut microbiome. Even more remarkable is that the transplantation of a phage, without bacteria, can have a physiological impact on the host. As is the case with conventional FMTs, if one expects the transfer of a beneficial phenotype from the donor, it is a reasonable possibility that an undesirable phenotype may also be transferred. Thus, it is as important to develop a mechanistic understanding of how phages influence the gut microbiome for improving therapeutic effect as it is to minimize adverse effects that are likely underreported.

## Phage-bacteria interactions

The healthy gut phageome is rich and diverse with correlations to health. The composition of phages in the human gut is highly individual-specific (Gregory et al., 2020; Shkoporov et al., 2019) and comprises virulent and temperate phages, with the latter predominant based on sequenced viral particles and the prevalence of prophages in gut bacteria. Recent work has revealed that healthy individuals possess a core set of virulent phages (Shkoporov et al., 2019). These phages are mostly non-enveloped DNA phages, either of the double-stranded DNA (dsDNA) *Caudovirales* or single-stranded DNA (ssDNA) *Microviridae* families (Manrique et al., 2016; Sausset et al., 2020), with RNA phages being comparatively rare (Zhang et al., 2006). The composition of the gut phageome has been linked to several diseases. For example, patients with IBD had significantly greater *Caudovirales* richness than in healthy controls, which coincided with decreased bacterial diversity and richness (Norman et al., 2015). Further analysis revealed that in contrast to the abundance of virulent phage in healthy individuals, patients with IBD had a virome largely composed of a temperate phage (Clooney et al., 2019). In ulcerative colitis, the altered mucosal virome is associated with a reduced diversity in function but greater representation of genes associated with bacterial fitness such as virulence and antimicrobial resistance (Zuo et al., 2018). Compositional links between the gut virome and host physiology has also been identified for malnutrition (Reyes et al., 2015), rheumatoid arthritis (Mangalea et al., 2021), type 1 and 2 diabetes (Ma et al., 2018; Tetz et al., 2019), Parkinson’s Disease (Tetz et al., 2018), and growth stunting (Khan Mirzaei et al., 2020).

Taxonomic classification of phage is challenging. Whereas early metagenomic studies have shed light on the complexity and richness of gut phages, much of the gut virome remains difficult to characterize and can neither be assigned a taxonomic position nor be linked to any microbial host (Aggarwala et al., 2017). In the absence of a universal phylogenetic marker (Roux et al., 2015), taxonomic assignment is typically limited to availability in reference databases, an issue that is being addressed with the expansion of the human Gut Phage Database (GPD) to more than 142,809 non-redundant phage genomes (Benler et al., 2021; Camarillo-Guerrero et al., 2021). Nevertheless, advances in culture-independent sequence-based analyses allowed the identification of thousands of phages belonging to three new families of the *Caudovirales* order (Benler et al., 2021), uncovered more than 10,000 viral species distributed over 248 viral families falling within 17 viral order-level clades (Shah et al., 2021, preprint), and improved our understanding of the human gut phage-host network by revealing new host-phage pairs (Džunková et al., 2019; Shah et al., 2021, preprint). The combined efforts of high-throughput sequencing and genomic data mining tools with cultureomics can lead to the identification, isolation, and purification of important phage species. The dsDNA phage crAssphage, named after the cross-assembly method by which it was first identified, is estimated to be the most abundant phage in the known human gut virome, comprising approximately 90% of VLP metagenomes (Dutilh et al., 2014). Since their initial discovery, crAss-like phages have been found to be relatively stable and widespread in the gut of virtually all tested individuals (Edwards et al., 2019; Manrique et al., 2016; Shkoporov et al., 2018). Culture-based assays have begun to more precisely identify their bacterial hosts, such as *Bacteroides intestinalis* (Shkoporov et al., 2018), *Bacteroides xylanisolvens* (Guerin et al., 2021), and *Bacteriodes thetaiotaomicron* (Hryckowian et al., 2020).

Phages have various lifestyles. In addition to the lytic life cycle that is pursued by virulent phages, temperate phages can also integrate their genomes into the bacterial chromosome as a prophage in the lysogenic life cycle. This expands the pool of bacterial genetic diversity beyond what is capable by mutation alone (Frazao et al., 2019). Metagenomic-sequencing has shown that prophages contribute to approximately 5% of the conserved function in the human gut microbiome (Qin et al., 2010) and are essential for the stability and maintenance of bacterial populations (Kim and Bae, 2018). These functions generally benefit the bacterial host by improving their competitive fitness in the GI environment by mechanisms that include their expansion of metabolic potential (Dragoš et al., 2021), improved survival (Brown et al., 2021), superinfection exclusion of phages (Bondy-Denomy et al., 2016), and environmental stress resistance (Wang et al., 2010). Some studies have suggested that phages are significant contributors to the spread of antibiotic resistance genes (Modi et al., 2013; Quirós et al., 2014), whereas others indicate that this is likely a rare occurrence in the human gut (Davies and Davies, 2010; Debroas and Siguret, 2019; Enault et al., 2017), especially when compared with conjugation (Džunková et al., 2019; Jiang et al., 2019). Prophage induction, leading to progeny phage that can spread to new hosts, can be initiated by various stimuli including gut metabolites (e.g., bile salts or nitric oxide) (Hernández et al., 2012), exogenous inducers such as quinolone antibiotics (Zhang et al., 2000), non-antibiotic medication (Sutcliffe et al., 2021), or specific dietary compounds (Boling et al., 2020; Oh et al., 2019). Prophage induction can also impact co-colonizing species as demonstrated by the production of the composite phage φV1/7 from *Enterococcous faecalis* V583 that lyses other competitor *E. faecalis* strains (Duerkop et al., 2012), or facilitate the release of intracellular bacteriocins (Colicin lb) as found with *Salmonella enterica* serovar Typhimurium targeting a competing commensal *Escherichia coli* (Nedialkova et al., 2016). As a result, prophage induction can contribute to gastrointestinal diseases by diminishing the ratio of symbionts to pathobionts, enabling pathogens to occupy the niche vacated by recently lysed commensals (Cornuault et al., 2018; Mills et al., 2013). Phage predation has also been observed to induce a type VIIb secretion system in *E. faecalis*, killing bystander species not directly targeted by the phage VPE25 (Chatterjee et al., 2021).

Phage predation can lead to bacterial resistance and phage counter effects. In some cases, bacteria do not develop genetic changes that confer phage resistance, possibly owing to phenotypic variation (Bull et al., 2014). In other cases, bacteria can remain completely susceptible to the phage by colonizing regions largely occluded from the phage access such deep in the intestinal mucosa. Outgrowth into the lumen may allow for those cells to become phage accessible and thus sustain phage propagation (Lourenço et al., 2020). However, the culture of a phage with its bacterial host often leads to a coexistence with phage-susceptible and phage-resistant bacterial species. Bacteria can develop resistance by a number of mechanisms including the inhibition of adsorption (receptor mutation, altered expression, or occlusion), interference with DNA injection (exclusion mechanisms), degradation of injected DNA (restriction modification or CRISPR-Cas systems), or abortive infection that prevents the spread of a progeny phage, among others (Houte et al., 2016). Whereas phage resistance may be beneficial against viral infection, the challenges associated with colonization is multifaceted, i.e., resistance development can result in a loss of beneficial functions (Gurney et al., 2020). This principle can be leveraged therapeutically. In *Pseudomonas aeruginosa*, resistance to the virulent OMKO1 phage results in a functionally defective outer membrane porin M, a crucial component in multidrug resistance leading to phage resistance but antibiotic susceptibility, or antibiotic resistance but phage susceptibility (Chan et al., 2016). Thus, the selective pressure provided by phage predation can attenuate virulence, termed “phage steering,” and has the potential to guide pathogenic bacteria to more therapeutically amenable situations (Gurney et al., 2020).

In response to the evolution of phage resistance, some phages are able to overcome such mechanisms by switching to other surface receptors on the same host (Meyer et al., 2012) or adapting to target other hosts (De Sordi et al., 2017). Phages are also able to counter bacterial nucleases by altering their genetic material to defend against restriction enzymes (Bair and Black, 2007), mutating sites targeted by CRISPR, or expressing anti-CRISPR proteins (ARCs) to either evade or directly inhibit host CRISPR-Cas systems (Bondy-Denomy et al., 2013).

## Phage interactions with the mammalian host

The mucosa of the human GI tract is essential for the maintenance of homeostasis, acting as an immunological and physiological barrier to infiltration by bacterial pathogens while harboring a rich population of commensal microorganisms (Allam-Ndoul et al., 2020; Vaga et al., 2020). Phages may have a significant role in this region as the T4 phage was shown to adhere to glycan residues in mucin via weak interactions with the immunoglobulin (Ig)-like domains of their capsid proteins, acting as a host-independent antimicrobial shield (Figure 1C) (Barr et al., 2013a, 2013b). This localization in the mucosa allows for sub-diffusive populations of phages to have an increased likelihood of host encounters (Barr et al., 2015). It is possible that this adherence strategy is utilized broadly as shown with Ig-like domains in the structural proteins of *Caudovirales* (Fraser et al., 2006) and *Bacteroides*-associated carbohydrate-binding (BACON) domains in crAssphages (De Jonge et al., 2019; Dutilh et al., 2014).

In addition to residing at the host-bacterial interface of the GI tract, phage particles are capable of directly interacting with the host (Bichet et al., 2021). Phages can be obtained from the GI tract through transcytosis (Nguyen et al., 2017) or permeation across a weakened intestinal barrier (Figure 1D) (Tetz and Tetz, 2016). Phages can also be carried across the epithelial barrier by dendritic cells when contained within bacteria (Figure 1E) (Duerkop and Hooper, 2013; Nguyen et al., 2017). Furthermore, epithelial and endothelial tissue may also act as a sink for exogenously administered phages (Bichet et al., 2021). Once accessed by the immune system, phages can act as immunomodulators, for example, by stimulating the production of anti-phage antibodies (Gogokhia et al., 2019; Kaźmierczak et al., 2021; Majewska et al., 2015, 2019), and possibly leading to a broader antiviral response (Duerkop and Hooper, 2013). In the case of *Enterococcus hirae*, a tape measure protein encoded by its prophage improved immunotherapy with cyclophosphamide through molecular mimicry of an oncogenic epitope, an effect that could be transferred to other commensal bacteria by heterologous expression (Fluckiger et al., 2020). Other studies by Gorski et al. suggested that phages can also exert an immunosuppressive protective role in the gut by acting with the immune system to control inflammatory and autoimmune reactions (Górski and Weber-Dabrowska, 2005). This was also shown with an *Enterococcus gallinarum* prophage ameliorating DSS-induced colitis in mouse models (Nishio et al., 2021). Contrarily, a study in a rodent model by Tetz and Tetz reported that the administration of a phage cocktail alters the composition of the intestinal microbiome and promotes loss of intestinal barrier integrity causing an increase in intestinal permeability (leaky gut) (Tetz and Tetz, 2016, 2018). Phages can also be pro-inflammatory as shown with the *E. coli* phage stimulating an inflammatory response in mice through the induction of interferon-gamma production by CD4^+^ T-cells in a Toll-like receptor dependent pathway, exacerbating colitis (Gogokhia et al., 2019; Gogokhia and Round, 2021). Another study reported that the internalization of the filamentous Pf phage virions, produced during chronic *P. aeruginosa* infection, may trigger a maladaptive antiviral response, leading to the suppression of bacterial clearance from infected wounds (Sweere Johanna et al., 2019).

## Precision modification of the gut microbiota

The potential role of commensal species in the gut as contributors to the development or exacerbation of human diseases has received considerable attention over the past decade. Duan et al. recently showed that the presence of cytolysin-producing *E. faecalis* correlates with the severity of liver disease and with mortality in patients with alcoholic hepatitis (Duan et al., 2019). Another study by Qi et al. reported an increase of *Bacteroides vulgatus* in the stool of individuals with polycystic ovary syndrome (PCOS). This effect could be induced in PCOS-like mouse models by FMT from women with PCOS or colonization with *B. vulgatus* (Qi et al., 2019). The specific nature of phage–bacterial interactions makes the use of phages an attractive strategy for the selective modification of individual species among a bacterial consortium. Applications of virulent phages against pathogens in phage therapy exemplify this strategy. In contrast, using a phage to target commensal species linked to disease allows one to potentially modulate host physiology without broadly altering the gut microbiota in a way that might occur with antibiotics. This was recently demonstrated with alcohol-related liver disease (ALD) in which a cocktail of virulent phages targeting cytolysin-producing *E. faecalis* abolished ALD in mice through bacterial knockdown (Duan et al., 2019). This indicates that the reduction of a commensal bacterium by a phage, and not necessarily its eradication, may be sufficient for the treatment of certain diseases.

In some cases, the introduction of a virulent phage can lead to effects beyond the species targeted. Using gnotobiotic mice colonized with a defined set of 10 human commensal bacteria and phages, longitudinal tracking of each microbe revealed that the knockdown of specific bacteria by their virulent phages altered the concentrations of co-colonizing species (i.e., those not directly targeted by phages) as well as the gut metabolome (Hsu et al., 2019). Such effects are not unexpected owing to the extensive cooperative and competitive interactions present in the gut microbiome (Coyte and Rakoff-Nahoum, 2019). The indirect effects of a lytic phage on non-host communities through metabolic and evolutionary mechanisms have been also reported by Fazzino et al., which showed that lysis of *E. coli* by the T7 phage increased *S. enterica* concentrations in coculture, owing to the released cell debris and the increased carbon secretion from *E. coli* cells that evolved resistance to the T7 phage (Fazzino et al., 2020).

**Figure 2:**
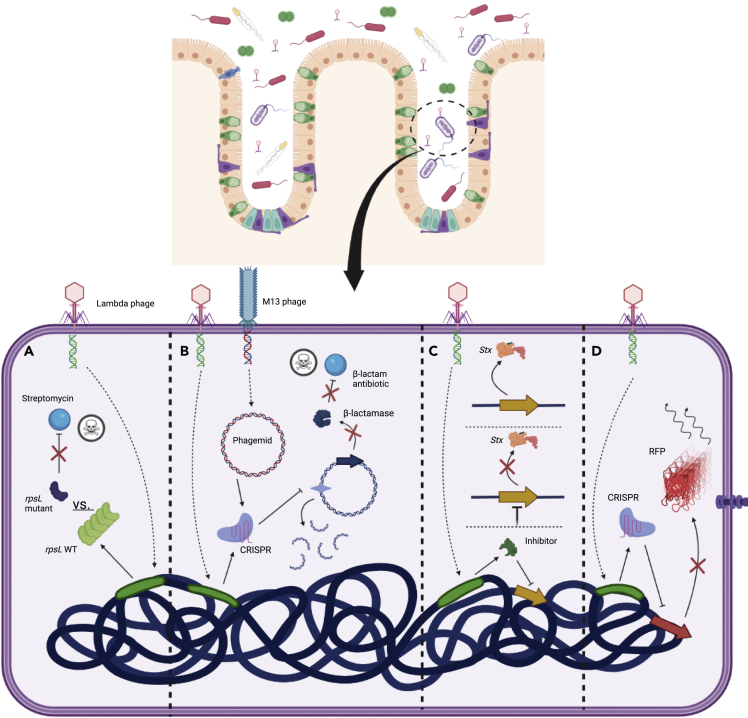
Modulation of bacterial function by genetically engineered phage (A) Over-expression of wildtype *rpsL* expressed from λ phage re-sensitizes the host bacterium to the antibiotic, streptomycin. (B) CRISPR-Cas elements expressed from a λ prophage or an M13 phagemid can re-sensitize bacteria to β-lactam antibiotics through the cleavage of plasmids containing antibiotic resistance genes (e.g., β-lactamases). (C) Expression of a transcriptional repressor for Shiga toxin (Stx) from a λ prophage can inhibit production of the toxin from bacteria colonizing the murine gut. (D) Expression of a nuclease inactivated Cas9 element from a λ prophage can be programmed to repress the expression of specific genes, such as a fluorescent marker protein, Red Fluorescent Protein (RFP).

A genetically engineered phage can sensitize bacteria to antibiotics. With the coming challenges associated with antibiotic resistance, developing strategies that improve their potency are important. Using an M13mp18 filamentous phage that produces a progeny phage without killing the host (i.e., the chronic phage cycle), over-expression of the *lexA3* repressor increased bacterial sensitivity to the antibiotics ofloxacin, gentamicin, and ampicillin (Lu and Collins, 2009). A temperate phage λ, encoding a multi-copy *rpsL* gene and “mock” *rpsL* gene containing silent mutations, re-sensitized a streptomycin-resistant *E. coli* to the antibiotic and decreased the minimum inhibitory concentration from 200 to 1.56 μg/mL (Figure 2A) (Edgar et al., 2012). To improve the efficacy of a virulent phage in combination with antibiotics, Yosef et al. engineered a λ phage to carry a CRISPR-Cas9 that targeted a plasmid-borne antibiotic resistance gene and T7 phage. Thus, lysogens would be re-sensitized to antibiotics but resistant to the T7 phage, whereas non-lysogens would remain susceptible to lysis by the T7 phage (Yosef et al., 2015). In a related approach, Citorik et al. used the M13 phage, engineered to express CRISPR-Cas9, to eliminate plasmid-borne resistance genes or to selectively kill antibiotic resistant bacteria within a mixed *E. coli* population (Figure 2B) (Citorik et al., 2014). Lam et al. used the M13 phage expressing CRISPR-Cas9 to target chromosomal fluorescent reporter genes in *E. coli* and found that escape mutants either lost phage-borne CRISPR-Cas9 components or mutated the targeted reporter gene (Lam et al., 2021). Using programmable nucleases to improve the potency or specificity of virulent phages as stand-alone antimicrobials is also an active area of research that has been well reviewed elsewhere (Lenneman et al., 2021).

Bacterial gene expression can be modulated by the phage within the mammalian gut. Of the breadth of functions expressed by bacteria, it may be that only a small subset is directly associated with disease. Exemplar are virulence factors that are present in some strains of *E. coli* but are not components of the core genome and are not found in commensal strains (Leimbach et al., 2013). Using an engineered phage to intracellularly modulate the expression of virulence factors substantially expands the possible targets beyond extracellular moieties that are typically the target of anti-virulence drugs (Dickey et al., 2017). Using a λ phage engineered to repress the enterohemorrhagic *E. coli* 933W prophage, this phage was shown to inhibit the production of Shigatoxin *in vitro* and *in vivo* (Figure 2C) (Hsu et al., 2020b). To broaden the versatility of this strategy, λ phage was engineered to express dCas9 with programmable RNAs for targeted gene repression. This was demonstrated with the specific knockdown of the RFP expression in *E. coli* colonizing the murine gut without substantially changing the fecal bacterial concentration (Figure 2D) (Hsu et al., 2020a).

## Summary and outlook

### Phage rehab

Phage therapy has an interesting history in leveraging a virulent phage as an antimicrobial strategy, one that is intertwined with antibiotics to elicit sometimes strong and often mixed opinions (Summers, 2012). In the early stages of modern medicine, when a bacterial infection could not be reliably treated, eradicating any and all bacteria was a boon to human health. Since then, our understanding of the mechanisms by which individual bacteria or their communities can contribute to health and disease has substantially improved, muddying the classification of some species as solely pathogenic or non-pathogenic especially without ecological context. Whereas the use of phages as antimicrobials is well described by phage therapy, it presumes a goal of bacterial eradication that is incompatible with emergent uses of phages in which treatment may not depend on bacterial eradication but instead the modulation of bacterial concentration or function, i.e., a rehabilitation of the gut microbiota.

In contrast to phage therapy, we suggest the use of the term “phage rehab” that would encompass the use of phages for the reduction or enrichment of bacterial species, or the modulation of one or multiple aspects of their function. As a result, the judicious application of the wildtype or engineered phage could be used to make subtle but therapeutically relevant changes in the mammalian gut. This would be distinct from phage steering, in which the desired outcome is the elimination of a bacterial pathogen.

### Specificity

In an environment saturated with substrates capable of redirecting phages by non-specific adsorption, their ability to find cognate bacteria and successfully propagate *in vivo* is a testament to their persistence and specificity. We have shown that the knockdown of specific bacteria can alter the concentration of species not-directly targeted within a defined consortium colonizing gnotobiotic mice (Hsu et al., 2019). Thus, well-designed cocktails of phages targeting extensively interacting species could have an amplified effect that is conducted to other co-colonizing species via inter-microbial interactions. Recent studies showed that a cocktail of coliphages, marketed as PreforPro, administered to healthy individuals altered the targeted gut *E. coli* as well as other bacterial species not directly targeted by the phage cocktail (Febvre et al., 2019; Grubb et al., 2020). The breadth and strength of this cascade effect will likely be dependent on the composition and density of co-colonizing microbes, as well as host type, health, and diet (Vujkovic-Cvijin et al., 2020). It will be especially crucial to have sufficient taxonomic resolution to track individual species, as alterations will be masked at a genus level or higher sequencing.

### A graded knockdown of bacteria

Targeting individual species with causal relationships to disease, such as cytolysin producing *E. faecalis* in ALD (Duan et al., 2019), suggests that therapeutic effect may be achieved by bacterial knockdown instead of complete elimination. An important consideration, which may not be easy to answer, is how much is enough? Duan et al. showed that ∼1-log reduction of *E. faecalis* was therapeutically sufficient in mouse models, which is promising. However, the bacterial response to a phage may vary depending on the co-colonizing species. Simplified *in vitro* cultures of *E. coli* and its phage (e.g., T5 or T7 phage) in minimal media showed only slight reductions in species concentration (<1 log) owing to the emergence of phage resistance, but in the presence of a nutrient competitor, *S. enterica*, there was a substantial reduction (>3-logs) (Harcombe and Bull, 2005). The converse, adding *E. coli* to a culture of *S. enterica* and its phage, Sf6 phage, did not have a similar effect, indicating the importance of the underlying mechanisms of resistance development, nutrient conditions, and competition (Harcombe and Bull, 2005).

### Intracellular access

Making genetic modifications to bacteria in the laboratory can be straightforward, provided the species is genetically tractable. However, once colonizing the gut, these bacteria become inaccessible by traditionally used techniques of genetic engineering. Injecting genetic material into their cognate bacteria is a process intrinsic to phage propagation and offers a potentially unique accessibility to bacteria in the mammalian gut. Using a temperate phage enables the durable modification with heterologous functions, targeting species colonizing the mammalian gut. In addition to phage-based mechanisms for microbial gene delivery, there are other interesting strategies based on conjugative plasmids (Neil et al., 2020; Ronda et al., 2019). Although these require direct contact with recipient cells and, in some cases, can have low efficiencies, they can be broadly disseminated across species, if desirable.

As we learn more about this microbial community colonizing the mammalian gut, an important next step is to develop strategies with commensurate sophistication that are capable of performing the desired modifications with precision and predictability. In a broad sense, these properties are natural aspects of phages, making them an attractive tool for use in rich and diverse ecosystems. Whether to provide mechanistic information through the specific probing of species or to make subtle compositional or function adjustments, the use of phages is an exciting and emergent area of research for the remodeling of the gut microbiome.
